# Bacterial isolates and antimicrobial susceptibility profiles of pediatric meningitis at comprehensive specialized hospitals in Bahir Dar, Northwest Ethiopia

**DOI:** 10.1371/journal.pone.0342467

**Published:** 2026-02-26

**Authors:** Tadese Sisay, Daniel Mekonnen, Mitkie Wendmagegn, Ayenew Berhan, Addisu Melese

**Affiliations:** 1 Department of Medical Laboratory Science, College of Health Sciences, Debre Tabor University, Debre Tabor, Ethiopia; 2 Department of Medical Laboratory Sciences, College of Medicine and Health Sciences, Bahir Dar University, Bahir Dar, Ethiopia; Debre Markos University, ETHIOPIA

## Abstract

Bacterial meningitis remains a major cause of morbidity and mortality among children in low-resource settings. Delayed diagnosis and antimicrobial resistance complicate effective management, and recent local data from Ethiopia are limited. This study aimed to determine the bacterial etiology and antimicrobial susceptibility patterns among children with suspected bacterial meningitis in Northwest Ethiopia. A hospital-based cross-sectional study was conducted from June 3 to December 25, 2024, among 189 pediatric patients aged 0–14 years who were clinically suspected of bacterial meningitis at two comprehensive specialized hospitals in Bahir Dar. Cerebrospinal fluid samples were collected aseptically and analyzed using standard cytological, biochemical, and microbiological methods. Antimicrobial susceptibility testing was performed using the Kirby–Bauer disk diffusion method in accordance with CLSI M100 (34th edition, 2024) guidelines. Data were entered and analyzed using SPSS version 26, and descriptive statistics, including frequencies and 95% confidence intervals for bacterial prevalence, were computed. Of the 189 cerebrospinal fluid samples analyzed, 10/189 (5.3%) were culture positive. Gram-negative bacteria accounted for 6/10 (60%) of isolates. *Escherichia coli* and *Staphylococcus aureus* were the most frequently identified pathogens, each representing 3/10 (30%), followed by *Klebsiella pneumoniae* at 2/10 (20%). Most confirmed cases occurred in infants aged 1–12 months. Ceftriaxone and imipenem were fully susceptible in *Staphylococcus aureus* and *Klebsiella pneumoniae*, while resistance was highest to ampicillin in *Escherichia coli* (2/3, 66.7%) and to ciprofloxacin in *Staphylococcus aureus* (1/3, 33.3%). Multidrug resistance among bacterial isolates was uncommon, with MDR detected in a single *Escherichia coli* isolate (33.3%), while no MDR was observed in other pathogens. In conclusion, Gram-negative bacteria predominated among pediatric bacterial meningitis cases in Northwest Ethiopia. These findings highlight the need for ongoing surveillance, improved laboratory capacity, and evidence-based antimicrobial stewardship to guide empirical therapy and improve pediatric outcomes.

## 1. Introduction

Meningitis remains a major public health concern, particularly among children, due to its rapid clinical progression, high fatality rate, and risk of permanent neurological sequelae. Globally, more than 2.5 million new cases and over 236,000 deaths from bacterial meningitis are reported each year, with children under five years disproportionately affected. The disease imposes a significant burden on health systems, families, and communities, especially in low- and middle-income countries [1,2].

Children are particularly vulnerable due to immature immune defenses, increasing disease susceptibility and severity. Case fatality can reach 70% in low-resource settings, and survivors often experience long-term complications, including cognitive impairment, hearing loss, and motor deficits [3,4].

The etiology of bacterial meningitis varies by age and geographic setting. In preterm neonates, *Escherichia coli* and Group B Streptococcus are the most common pathogens, while term neonates and infants under three months are mainly affected by Group B Streptococcus, *Escherichia coli*, *Streptococcus pneumoniae*, and *Listeria monocytogenes*. In children aged three months to ten years, *Streptococcus pneumoniae*, *Neisseria meningitidis*, and *Haemophilus influenzae* predominate. Gram-negative bacteria are increasingly reported in resource-limited settings [5,6].

Bacterial meningitis among children remains a major public health burden in sub-Saharan Africa, including Ethiopia, due to recurrent outbreaks, limited diagnostic capacity, delayed healthcare access, malnutrition, incomplete immunization, overcrowding, and widespread empirical antibiotic use [7,8]. Ethiopia remains among the countries with persistently high rates of childhood meningitis-related hospitalization and mortality, with reported hospitalization rates ranging from 6% to 8% and case fatality rates reaching up to 33.6% [2,7]. Malnutrition, incomplete vaccination, overcrowding, and empirical antibiotic use exacerbate disease burden [9,10].

Epidemiology varies by age, region, and vaccination status [5]. Although *Streptococcus pneumoniae*, *Neisseria meningitidis*, and *Haemophilus influenzae* historically dominated, conjugate vaccines have shifted pathogen distribution [4,11,12]. Gram-negative bacteria like *Escherichia coli* and *Klebsiella pneumoniae* remain important in neonates and young infants, highlighting the need for ongoing local surveillance [13–15].

Antimicrobial resistance (AMR) further complicates the management of acute bacterial meningitis (ABM) in children and represents a growing global public health threat. In 2019 alone, AMR was directly responsible for an estimated 1.27 million deaths worldwide [16]. In Ethiopia, empirical treatment of suspected meningitis is common due to limited access to culture and antimicrobial susceptibility testing, increasing the risk of inappropriate therapy, treatment failure, and the emergence of multidrug-resistant (MDR) pathogens [9,17,18]. The rising prevalence of MDR organisms among common meningitis pathogens poses a serious challenge to existing treatment guidelines and threatens to reverse gains made through vaccination and improved clinical care [15,19,20].

Despite the high burden in Ethiopia, current local data on bacterial etiology and antimicrobial susceptibility are scarce, especially in Northwest Ethiopia. Most previous studies were single-center, limited, or pre-vaccine, reducing relevance [7,9,13,21]. Furthermore, local AMR data necessary to inform empirical therapy and antimicrobial stewardship remain scarce [17,20]. This study aimed to identify bacterial isolates and their antimicrobial susceptibility patterns among children suspected of bacterial meningitis in Bahir Dar, Northwest Ethiopia using CLSI-guided laboratory methods.

## 2. Materials and methods

### 2.1. Study design, area, and period

A hospital-based cross-sectional study was conducted from June 3, 2024 to December 25, 2024, encompassing both participant recruitment and data collection, at Felege Hiwot Comprehensive Specialized Hospital (FHCSH) and Tibebe Ghion Specialized Teaching Hospital (TGSTH) in the Amhara National Regional State (ANRS), Northwest Ethiopia.

The ANRS is located in northern Ethiopia and includes both highland and lowland areas. FHCSH and TGSTH are tertiary-level referral hospitals that together provide specialized healthcare services to an estimated catchment population exceeding 7 million people.

### 2.2. Study population

The study population consisted of pediatric patients aged ≤14 years who presented to comprehensive specialized hospitals in Bahir Dar, Northwest Ethiopia, during the study period with clinical features suggestive of bacterial meningitis. Participants were identified during routine clinical care following standard medical history taking, physical examination, and initial laboratory evaluation.

### 2.3. Eligibility criteria

#### 2.3.1. Inclusion criteria.

Children were eligible for inclusion if they met all of the following criteria:

- Age ≤ 14 years
- Presentation with sudden-onset fever (>38.5°C rectal or >38.0°C axillary)
- Presence of at least one meningeal sign, including neck stiffness, altered level of consciousness, bulging fontanelle (in infants), or other signs of meningeal irritation such as photophobia or vomiting [18,22].
- Underwent lumbar puncture with cerebrospinal fluid (CSF) samples submitted for laboratory analysis

#### 2.3.2. Exclusion criteria.

Children were excluded from the study if they met any of the following conditions:

- CSF specimens that were insufficient in volume, contaminated, or improperly collected
- Incomplete clinical, sociodemographic, or laboratory records

### 2.4. Sample size determination and sampling technique.

A convenience sampling technique was employed, whereby all eligible pediatric patients who met the inclusion criteria were enrolled consecutively during the study period at the two hospitals. The sample size was estimated using a single population proportion formula:


$$n\;=\;{\left(Z\alpha /\text{2}\right)}^{\text{2}}\;P\;(\text{1}\;-\;P)/d^{\text{2}}$$

where n is the required sample size, Z is the standard normal value at a 95% confidence level (1.96), p is the estimated prevalence of bacterial meningitis (13.2%) based on a previous study conducted at Debre Markos Hospital [23], and d is the margin of error (5%). Using this formula, the calculated minimum sample size was 189.

The total sample was proportionally allocated between Felege Hiwot Comprehensive Specialized Hospital (FHCSH) and Tibebe Ghion Specialized Teaching Hospital (TGSTH), with approximately 94 and 95 cerebrospinal fluid (CSF) samples collected, respectively. Minor variations in the number of samples between sites did not significantly affect bacterial isolation rates, and comparable positivity rates were observed across the two hospitals.

### 2.5. Data collection and processing

#### 2.5.1. Sociodemographic and clinical data collection.

Sociodemographic and clinical information was collected from parents or guardians using a pretested structured questionnaire administered through face-to-face interviews by trained health professionals. Relevant clinical data, including presenting symptoms and prior antibiotic exposure, were obtained from medical records, while laboratory findings were recorded from CSF analysis results using a standardized data extraction checklist.

#### 2.5.2. Laboratory data collection and processing.

**2.5.2.1. CSF collection and processing:** Cerebrospinal fluid (CSF) specimens were collected by experienced physicians via lumbar puncture under strict aseptic conditions, in accordance with standard CSF analysis and meningitis diagnostic guidelines. Immediately after collection, the volume and gross appearance of the CSF (clear, cloudy, bloody, or xanthochromic) were recorded. Specimens were transported without delay to the Medical Microbiology Laboratory at Bahir Dar University and CSF specimens were processed promptly, typically within 15–30 minutes, and no later than two hours after collection.

Total white blood cell (WBC) count and differential count were determined using standard hemocytometer methods. CSF specimens were inoculated onto blood agar, chocolate agar, MacConkey agar, and Mannitol Salt Agar (MSA). Blood and chocolate agar plates were incubated at 36°C in a candle jar for 24–48 hours, while MacConkey and MSA plates were incubated aerobically at 37°C. MSA was used specifically to differentiate *S. aureus* (yellow colonies) from coagulase-negative Staphylococci (pink/red colonies). Culture plates were examined after 24 hours, and those showing no visible growth were re-incubated and re-examined at 48 hours before being reported as negative.

**2.5.2.2. Bacterial isolation and identification:** Presumptive bacterial identification was based on colony morphology, hemolysis pattern, pigment production, mannitol fermentation (MSA), and other phenotypic characteristics. Gram staining followed by conventional biochemical tests including catalase, coagulase, oxidase, urease, indole, citrate utilization, carbohydrate fermentation tests, and optochin susceptibility were performed for genus- and species-level identification, in accordance with standard microbiological procedures [24].

**2.5.2.3. Antimicrobial susceptibility testing:** Antimicrobial susceptibility testing was performed using the Kirby–Bauer disk diffusion method on Mueller–Hinton agar (MHA), with interpretations based on CLSI M100, 34th edition (2024) [25]. Only antibiotics recommended for routine testing of pediatric meningitis pathogens were included. Ciprofloxacin was tested for surveillance purposes only and is not considered a first-line agent in children. Chloramphenicol was included solely for *Haemophilus influenzae* and *Streptococcus pneumoniae*, reflecting its historical use in pediatric meningitis treatment. Ampicillin testing was excluded for *Klebsiella pneumoniae* due to intrinsic resistance mediated by chromosomal SHV-1 β-lactamase. AST for *Streptococcus pneumoniae* and *Haemophilus influenzae* was performed on MHA supplemented with 5% sheep blood.

Bacterial suspensions were adjusted to a 0.5 McFarland standard and inoculated onto agar plates. Antibiotic discs were applied using sterile forceps at 15 mm spacing on 150 mm plates, followed by incubation at 37°C for 18–24 hours. Inhibition zones were interpreted according to CLSI 2024 breakpoints. Multidrug resistance (MDR) was defined as resistance to three or more antibiotic classes among tested antibiotics, in line with CLSI 2024 guidance. MDR evaluation included only antibiotics tested for each species according to the recommended panels [25].

### 2.6. Data quality assurance

Quality assurance procedures were implemented throughout the pre-analytical, analytical, and post-analytical phases in accordance with standard operating procedures. Prior to data collection, a site assessment and pretest were conducted to ensure the functionality of laboratory procedures. Data collectors and laboratory personnel received standardized training on specimen handling and data collection.

All cerebrospinal fluid (CSF) specimens were checked for proper labeling, adequate volume, sterility, and timely transport. Samples that were grossly blood-contaminated, delayed beyond two hours, or improperly transported were excluded; however, all 189 CSF samples met the predefined quality criteria and were included in the analysis.

Laboratory analyses were performed by trained and experienced personnel. The sterility and performance of culture media and antimicrobial susceptibility testing were verified using standard ATCC reference strains, including *Escherchi coli* ATCC 25922, *Staphylococcus aureus* ATCC 25923, and *Streptococcus pneumoniae* ATCC 49619, in accordance with CLSI guidelines.

### 2.7. Data analysis and interpretation

Data were entered, cleaned, and analyzed using SPSS version 26. Descriptive statistics, including frequencies, proportions, and cross-tabulations, were used to summarize sociodemographic, clinical, and laboratory characteristics. The prevalence of bacterial meningitis was calculated with 95% confidence intervals, and results were presented using tables and figures. All analyses were conducted at a 95% confidence level.

### 2.8. Ethical consideration

Ethical clearance was obtained from the Institutional Review Board (IRB) of the College of Medicine and Health Sciences, Bahir Dar University (Reference number: CMHS 1039/2024). The IRB reviewed and approved the use of verbal informed consent due to the high proportion of illiterate guardians among the study population. Accordingly, verbal consent was obtained from all parents or legal guardians, and children provided assent when appropriate. For literate guardians, written informed consent was obtained. The consent process was conducted by trained data collectors and verified by supervisors, and all participant consents were documented as per IRB-approved procedures. In order to anonymize study participants, data collection was done through coding. Confidentiality was maintained for clinical results. The study procedures adhered to the principles outlined in the Declaration of Helsinki. Support letters were acquired from each hospital administration and the Amhara Public Health Institute based on the ethical clearance.

## 3. Results

### 3.1. Socio-demographic and clinical characteristics of children

**Fig 1:**
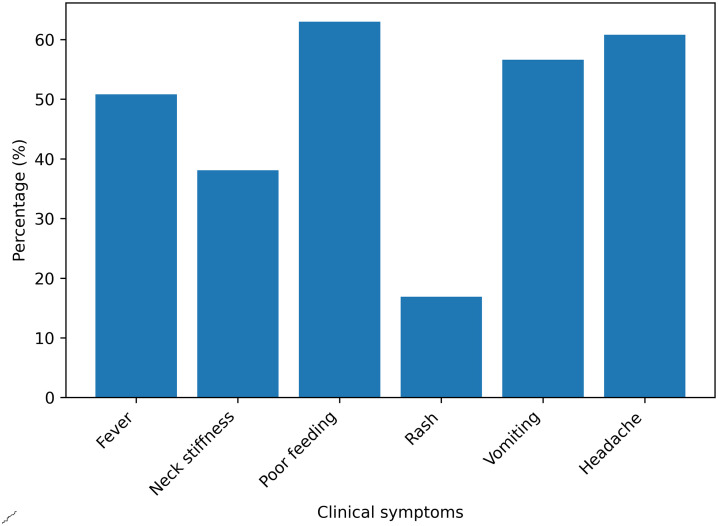
Clinical sign and symptom of study participants at FHCSH and TGTH, North West Ethiopia, 2024.

A total of 189 children with presumptive bacterial meningitis were enrolled, of whom 89 (47.1%) were male. Participants’ ages ranged from 2 days to 14 years, with a median age of 240 days (~8 months) and an interquartile range (IQR) of 25–1460 days (~4 years). Most participants, 103 (54.5%), were from rural areas. Nutritional status was assessed using weight-for-age Z-scores (WAZ) according to WHO Child Growth Standards. 38 children (20.1%) were classified as underweight (WAZ <−2 SD) (Table 1). The most frequent clinical complaints were loss of appetite (63%) and headache (60.8%), whereas skin rash was the least observed sign (Fig 1).

**Table 1:** Socio-demographic and clinical characteristics of children at FHCSH and TGSTH, Northwest Ethiopia, 2024.

Variable	Category	Frequency (%)	Culture positive N (%)
Sex	Male	89 (47.1)	5 (5.6)
	Female	100 (52.9)	5 (5)
Age	≤28 days	53 (28.0)	2 (3.7)
	28 day–12 months	65 (34.4)	4 (6.1)
	1–5 years	34 (18)	2 (5.8)
	6–10 years	23 (12.2)	1 (4.3)
	11–14 years	13 (6.9)	1 (7.6)
Residence	Urban	86 (45.5)	4 (4.6)
	Rural	103 (54.5)	6 (5.8)
Mother’s education	Cannot read/write	69 (36.5)	6 (8.6)
	Primary	60 (31.7)	1 (1.6)
	Secondary	35 (18.5)	2 (5.7)
	Diploma & above	25 (13.2)	1 (4)
Mother’s occupation	Farmer	99 (52.4)	6 (6)
	Housewife	49 (25.9)	1 (2)
	Merchant	32 (16.9)	2 (6)
	Other*	9 (4.8)	1 (11)
House partitions	≤4	92 (48.7)	6 (6.5)
	>4	97 (51.3)	4 (4.1)
People sharing room	≤2	73 (38.6)	2 (2.7)
	3–4	102 (54)	4 (3.9)
	>4	14 (7.4)	4 (28.5)
Mode of delivery	Spontaneous vaginal	104 (55)	3 (2.8)
	Assisted vaginal	75 (39.7)	5 (6.6)
	Cesarean section	10 (5.3)	2 (20)
Duration of illness	<1 week	94 (49.7)	3 (3.1)
	>1 week	95 (50.3)	7 (7.3)
Tonsillectomy	Yes	49 (25.9)	7 (14.2)
	No	140 (74.1)	3 (2.1)
Vaccination	Fully vaccinated	143 (75.7)	7 (4.8)
	Incompletely vaccinated	46 (24.3)	3 (6.5)
Admission history	Yes	80 (42.3)	6 (7.5)
	No	109 (57.7)	4 (3.6)
Previous drug use	Yes	34 (18)	1 (2.9)
	No	155 (82)	9 (5.8)
Comorbidity	Yes	78 (41.3)	5 (6.4)
	No	111 (58.7)	5 (4.5)
Underweight	Yes	38 (20.1)	6 (15.7)
	No	151 (79.9)	4 (2.6)
Maternal UTIs/STIs	Yes	34 (18)	1 (2.9)
	No	155 (82)	9 (5.8)

*Other = Civil servant, daily labor, no work.

### 3.2. CSF cell count and biochemical analysis

The mean CSF white blood cell (WBC) count in confirmed bacterial meningitis was 1,240 cells/µL (range: 420–2,500 cells/µL), with a predominance of neutrophils (>80%). In contrast, non-meningitis cases had a mean WBC count of 45 cells/µL (range: 0–150 cells/µL) (Table 2). Bloody CSF samples were interpreted cautiously, and culture results were correlated with clinical findings.

**Table 2:** CSF findings among children with suspected meningitis at FHCSH and TGSTH, Northwest Ethiopia, 2024.

CSF Parameter	Confirmed BM (n = 10)	Non-meningitis (n = 179)
WBC count (cells/µL)	1,240 (420–2,500)	45 (0–150)
Neutrophil %	>80%	<30%
Glucose (mg/dL)	28 (10–40)	58 (40–75)
Protein (mg/dL)	210 (120–380)	55 (25–90)
CSF characteristics	Clear: 136 (72)	Clear: 3 (2.2)
	Yellow: 27 (14.3)	Yellow: 2 (7.4)
	Bloody: 12 (6.3)	Bloody: 1 (8.3)
	Turbid: 14 (7.4)	Turbid: 4 (28)

### 3.3. Prevalence of bacterial isolates

**Fig 2:**
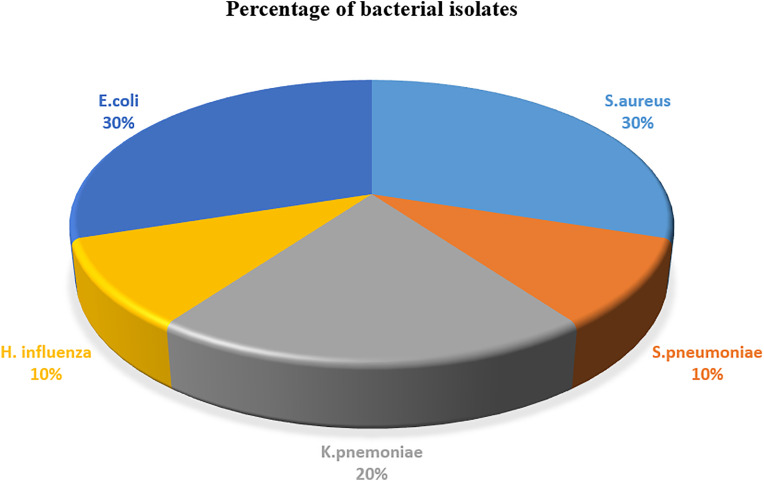
Types and frequency of bacterial isolates from CSF cultures of study participants at FHCSH and TGTH, North West Ethiopia, 2024.

Of the 189 CSF samples processed, 10 (5.3%; 95% CI: 2.6–8.5) were positive for bacterial meningitis. Five bacterial species were recovered, with Gram-negative bacteria predominating (60%). *Staphylococcus aureus* and *Escherchi coli* were most common, each representing 30% of isolates, followed by *Klebsiella pneumoniae* (20%). No mixed infections were observed (Fig 2).

Distribution of isolates by demographics showed a slightly higher prevalence among rural children (5.8%) than males (5.6%). Most isolates (40%) occurred in children aged 28 days–12 months. Children with prior hospitalization had higher prevalence (7.5%) compared to those with previous antibiotic use (3%) (Table 3).

**Table 3:** Distribution of bacterial isolates among children with suspected meningitis at TGSTH and FHCSH, Northwest Ethiopia, 2024.

Variable	Category	Total positive (n)	*Escherchi coli*	*Staphylococcus aureus*	*Klebsiella pneumoniae*	*Streptococcus pneumoniae*	*Haemophilus influenzae*
Sex	Male	5	1 (20%)	2 (40%)	1 (20%)	1 (20%)	0
	Female	5	2 (40%)	1 (20%)	1 (20%)	0	1 (20%)
Age	<28 days	2	1 (50%)	0	1 (50%)	0	0
	28 days–12 months	4	1 (25%)	2 (50%)	1 (25%)	0	0
	1–14 years	4	1 (25%)	1 (25%)	0	1 (25%)	1 (25%)
Residence	Urban	4	0	2 (50%)	1 (25%)	1 (25%)	0
	Rural	6	3 (50%)	1 (17%)	1 (17%)	0	1 (17%)
Mother’s education	Cannot read/write	6	2 (33%)	2 (33%)	2 (33%)	0	0
	Primary	1	1 (100%)	0	0	0	0
	Secondary	2	0	0	0	1 (50%)	1 (50%)
	Diploma & above	1	0	1 (100%)	0	0	0
Mother’s occupation	Farmer	6	2 (33%)	3 (50%)	1 (17%)	0	0
	Housewife	1	1 (100%)	0	0	0	0
	Merchant	2	0	0	0	1 (50%)	1 (50%)
	Other	1	0	0	1 (100%)	0	0

### 3.4. Antimicrobial susceptibility testing

Among CSF isolates, *Escherchi coli* was 2/3 (66.6%) susceptible to ceftriaxone, 3/3 (100%) to imipenem, and 1/3 (33.3%) resistant to ciprofloxacin. *Klebsiella pneumoniae* was fully susceptible to ceftriaxone and imipenem, with 1/2 (50%) resistance to ciprofloxacin. *Staphylococcus aureus* was fully susceptible to ceftriaxone and imipenem, and 2/3 (66.6%) susceptible to trimethoprim-sulfamethoxazole and ciprofloxacin. *Haemophilus influenzae* was susceptible to all tested agents, and *Streptococcus pneumoniae* was susceptible to trimethoprim-sulfamethoxazole but resistant to chloramphenicol (Table 4).

**Table 4:** Antimicrobial susceptibility profile of bacterial isolates from CSF in pediatric meningitis, Northwest Ethiopia, 2024.

Organism	Antibiotic tested	Susceptible n (%)	Resistant n (%)
*Staphylococcus aureus* (n = 3)	Ciprofloxacin	2 (66.7)	1 (33.3)
	Ceftriaxone	3 (100)	0 (0)
	Trimethoprim-sulfamethoxazole	2 (66.7)	1 (33.3)
	Imipenem	3 (100)	0 (0)
*Escherchi coli* (n = 3)	Ampicillin	1 (33.3)	2 (66.7)
	Ceftriaxone	2 (66.7)	1 (33.3)
	Ciprofloxacin	2 (66.7)	1 (33.3)
	Imipenem	3 (100)	0 (0)
*Klebsiella pneumoniae* (n = 2)	Ceftriaxone	2 (100)	0 (0)
	Ciprofloxacin	1 (50)	1 (50)
	Imipenem	2 (100)	0 (0)
*Haemophilus influenzae* (n = 1)	Ceftriaxone	1 (100)	0 (0)
	Chloramphenicol	1 (100)	0 (0)
	Ciprofloxacin	1 (100)	0 (0)
	Trimethoprim-sulfamethoxazole	1 (100)	0 (0)
*Streptococcus pneumoniae* (n = 1)	Chloramphenicol	0 (0)	1 (100)
	Trimethoprim-sulfamethoxazole	1 (100)	0 (0)

†Ciprofloxacin results are reported for epidemiological surveillance only and are not recommended for empirical therapy in pediatric meningitis.

### 3.5. Multidrug resistance patterns

Multidrug resistance was detected among a limited number of cerebrospinal fluid bacterial isolates. Among Gram-negative bacteria, 1 of 6 isolates (16.7%), specifically *Escherchi coli*, met the MDR criteria, exhibiting concurrent resistance to ampicillin, ceftriaxone, and ciprofloxacin. No MDR was observed in *Staphylococcus aureus*, *Klebsiella pneumoniae*, *Haemophilus influenzae*, or *S. pneumoniae* isolates (Table 5).

**Table 5:** Multidrug resistance profile of bacterial isolates from CSF in pediatric meningitis, Northwest Ethiopia, 2024.

Bacterial species	Number tested	MDR n (%)	Resistance pattern
*Staphylococcus aureus*	3	1 (33.3)	Not MDR
*Escherchi coli*	3	1 (33.3)	Ampicillin + Ceftriaxone + Ciprofloxacin
*Klebsiella pneumoniae*	2	0 (0.0)	Not MDR
*Haemophilus influenzae*	1	0 (0.0)	Not MDR
*Streptococcus pneumoniae*	1	0 (0.0)	Not MDR

## 4. Discussion

Overall, bacterial meningitis was confirmed in 5.3% of suspected pediatric cases, predominantly affecting infants under one year and children from rural areas. Gram-negative bacteria accounted for the majority of isolates (60%), with *Staphylococcus aureus* and *Escherchi coli* each representing 30% of the recovered pathogens. Multidrug resistance among bacterial isolates was uncommon, with MDR detected in only one *Escherchi coli* isolate (33.3%). These findings highlight the continued clinical relevance of bacterial meningitis and the importance of local antimicrobial resistance surveillance to inform empirical therapy.

Bacterial meningitis remains a significant cause of morbidity and mortality in children, especially in resource-limited settings like Ethiopia, despite the availability of vaccines [13,22]. Early diagnosis, appropriate empirical therapy, and vaccination remain essential to reduce disease burden. However, the effectiveness of empirical treatment depends heavily on reliable local antimicrobial susceptibility data generated using standardized methods [15,26].

In this study, the overall bacterial isolation rate was 5.3% (95% CI: 2.6–8.5%), comparable to previous reports from Hawassa (8.5%) [27], but lower than rates reported in India (28%) and other Ethiopian regions such as Dilla (13.2%) [7]. Low culture positivity may reflect prior antibiotic exposure and limited sensitivity of conventional methods, highlighting the need for molecular diagnostics [20].

Regionally, our isolation rate was higher than those reported in Gondar (1.7%) and Ghana (0.8%) [11,13], reflecting variability in healthcare access, diagnostic capacity, and sample handling. Bacterial meningitis was most prevalent in children under one year, consistent with prior studies highlighting the vulnerability of neonates and young infants due to immunological immaturity [7,14,28]. Higher prevalence among rural children underscores healthcare inequities and delayed access to treatment [29].

Gram-negative bacteria predominated (60%), with *Staphylococcus aureus* and *Escherchi coli* each accounting for 30% of isolates, followed by *Klebsiella pneumoniae* (20%). These findings are consistent with reports from Bahir Dar [21] and Nepal (72.2%) [30]. The predominance of gram-negative organisms may be influenced by maternal colonization, hygiene practices, and healthcare exposure. Low detection of *Haemophilus influenzae* and *Streptococcus pneumoniae* likely reflects vaccine coverage, consistent with WHO surveillance and prior Ethiopian studies [7,12].

Regarding AMR, *Staphylococcus aureus* showed only 33.3% resistance to ciprofloxacin, while remaining fully susceptible to ceftriaxone and imipenem, consistent with regional data [21]. *Escherichia coli* showed 66.6% resistance to ampicillin, 33.3% resistance to ceftriaxone and ciprofloxacin, and complete susceptibility to imipenem, findings that are consistent with reports from China, Ethiopia, and Iran [12,31]. Although *Klebsiella pneumoniae* isolates were fully susceptible to ceftriaxone and imipenem in this study, global reports of ESBL-producing strains highlight the need for continued investigation [32]. The single *Streptococcus pneumoniae* isolate was resistant to chloramphenicol, whereas the *Haemophilus influenzae* isolate remained susceptible to all tested antibiotics. Despite the small number of isolates, these findings emphasize the importance of ongoing local and global AMR monitoring [33,34].

In this study, multidrug resistance among bacterial isolates causing pediatric meningitis was uncommon, with MDR detected in a single *Escherchi coli* isolate (33.3%) and no MDR observed in other pathogens. Although overall MDR was low, similar studies in Ethiopia have reported substantial resistance among meningitis pathogens, particularly Gram-negative bacteria, highlighting a significant AMR burden in this context. These findings underscore the potential risk of treatment failure if empirical therapy is not guided by local susceptibility patterns. Strengthening national antimicrobial stewardship programs is therefore critical, particularly in rural areas where antibiotic access is common and misuse remains prevalent [20,32].

### 4.1. Limitations of the study

This study has limitations. The hospital-based consecutive sampling may limit the generalizability of the findings, prior antibiotic exposure might have reduced culture yield, and molecular diagnostic tests were unavailable. Antimicrobial susceptibility testing was limited to disk diffusion without minimum inhibitory concentration (MIC) determination. Despite these limitations, the study provides essential local data to guide pediatric meningitis treatment in Ethiopia.

## 5. Conclusion

This study confirms Gram-negative bacteria, including *Escherchi coli* and *Klebsiella pneumoniae*, predominated, with most isolates susceptible to ceftriaxone and imipenem. Antimicrobial resistance among both Gram-positive and Gram-negative isolates poses significant challenges. Continuous local surveillance, strengthened laboratory capacity, and antimicrobial stewardship are essential to guide effective empirical therapy and reduce disease burden.

## Supporting information

S1 Table Data on AST of CSF bacterial isolates. (DOCX)

## Abbreviations

AMR: Antimicrobial Resistance
CLSI: Clinical Laboratory Standards Institute
CSF: Cerebrospinal Fluid
FHCSH: Felege Hiwot Comprehensive Specialized Hospital
MDR: Multidrug-Resistant
MIC: Minimum Inhibitory Concentration
UTI: Urinary tract infection
TGSTH: Tibebe Ghion Specialized Teaching Hospital.
