# Supplementary material for: Bacterial isolates and antimicrobial susceptibility profiles of pediatric meningitis at comprehensive specialized hospitals in Bahir Dar, Northwest Ethiopia
Source: PLoS One. 2026 Feb 26;21(2):e0342467. doi: 10.1371/journal.pone.0342467 (PMC12944789; doi:10.1371/journal.pone.0342467)
Supplement: S1 Table — (DOCX) [file pone.0342467.s001.docx]

**Title:**

Antimicrobial susceptibility data of CSF bacterial isolates from pediatric meningitis patients in Northwest Ethiopia

| No | Isolated Organism | Antibiotics | | | | | |
| --- | --- | --- | --- | --- | --- | --- | --- |
|  |  | **Ampicillin** | **Ceftriaxone** | **Ciprofloxacin** | **Imipenem** | **Trimethoprim-sulfamethoxazole** | **Chloramphenicol** |
| 1 | ***Staphylococcus aureus*** | NA | S | R | S | S | NA |
| 2 | ***Staphylococcus aureus*** | NA | S | S | S | R | NA |
| 3 | ***Staphylococcus aureus*** | NA | S | S | S | S | NA |
| 4 | ***Escherichia coli*** | R | S | S | S | NA | NA |
| 5 | ***Escherichia coli*** | R | R | R | S | NA | NA |
| 6 | ***Escherichia coli*** | S | S | S | S | NA | NA |
| 7 | ***Klebsiella pneumoniae*** | NA | S | R | S | NA | NA |
| 8 | ***Klebsiella pneumoniae*** | NA | S | S | S | NA | NA |
| 9 | ***Haemophilus influenzae*** | NA | S | S | NA | S | S |
| 10 | ***Streptococcus pneumoniae*** | NA | NA | NA | NA | S | R |

Key:

S = Susceptible

R = Resistant

NA = Not tested

Notes: The dataset is fully anonymized and contains no personal or clinical identifiers.
